# Genetic Diversity and Phylogeny of the Genus *Euplotes* (Protozoa, Ciliophora) Revealed by the Mitochondrial CO1 and Nuclear Ribosomal Genes

**DOI:** 10.3390/microorganisms9112204

**Published:** 2021-10-22

**Authors:** Congcong Wang, Yue Hu, Alan Warren, Xiaozhong Hu

**Affiliations:** 1 Key Laboratory of Mariculture, Ministry of Education, College of Fisheries, Ocean University of China, Qingdao 266003, China; wangcocoh@163.com; 2 Oxford Sixth Form College, Oxford OX1 4HT, UK; yue.hu@oxfordsixthformcollege.com; 3 Department of Life Sciences, Natural History Museum, London SW7 5BD, UK; a.warren@nhm.ac.uk

**Keywords:** ciliate, CO1 gene, genetic diversity, microeukaryote, molecular phylogeny, SSU rDNA

## Abstract

Nuclear ribosomal and mitochondrial genes have been utilized individually or in combination to identify known species and discriminate closely related species. However, compared with metazoans, genetic diversity within the ciliate order Euplotida is poorly known. The aim of this study is to investigate how much nucleotide sequence divergence occurs within *Euplotes*. A total of 14 new gene sequences, comprising four SSU rDNA and 10 CO1 (including three species for the first time) were obtained. Phylogenetic analyses were carried out based on sequences of two DNA fragments from the same 27 isolates. We found that CO1 revealed a larger interspecific divergence than the SSU rRNA gene, thus demonstrating a higher resolution for separating congeners. Genetic distances differ significantly at the species level. *Euplotes balteatus* was revealed to have a large intraspecific variation at two loci, while *E. vannus* showed different levels of haplotype variability, which appeared as a polyphyletic cluster on the CO1 tree. These high genetic divergences suggest the presence of more cryptic species. By contrast, the CO1 gene showed low variability within *E. raikovi*, appearing as monophyletic clusters, which indicates that this species could be identified based on this gene. Conclusively, CO1 is a suitable marker for the study of genetic diversity within *Euplotes*, and increased taxon sampling gives an opportunity to screen relationships among members of this genus. Additionally, current data present no clear biogeographical pattern for *Euplotes*.

## 1. Introduction

Traditional taxonomy of protozoans relies heavily on high expertise. Numerous studies have suggested a higher protozoan diversity than originally proposed [1], which brings a great challenge for taxonomy. Therefore, it is critical to seek technical assistance for the initial description and subsequent recognition of organisms [2]. Though a gene-based identification system can aid the resolution of an organism’s diversity, the identification and determination of the systematic position of a species using genetic information is still a tempting target. Ciliates (phylum Ciliophora), as a diverse group of protozoans, have a wide geographical distribution and play a vital ecological role as trophic links in microbial food webs [3,4,5]. Despite this significance, our knowledge of ciliate genetic diversity is neither complete nor equal across the entire phylum, especially when compared with the huge amount of morphological information. To date, a variety of molecular methods (e.g., DNA–DNA hybridization, gene sequencing, and RAPD fingerprinting) have been used to assess genetic variation at different taxonomic levels [3,6,7]. Among these, comparisons of nuclear small-subunit ribosomal RNA gene (SSU rDNA) sequences are commonly applied [8,9,10,11,12,13,14]. However, recent studies have shown the limitations of SSU rDNA for discriminating closely related congeners [15,16,17,18]. Another genetic marker is, therefore, needed.

Compared with nuclear DNA, the evolution of the cytochrome *c* oxidase subunit I (CO1) gene is fast enough to allow the discrimination of not only closely related organisms, but also phylogeographic groups within a single species [19]. Folmer et al. (1994) [20] conducted a pilot study to design “universal” DNA primers for a polymerase chain reaction (PCR) amplification of the CO1 gene in 11 metazoan invertebrate phyla. Thereafter, CO1 sequence divergences have been used to discriminate closely related species in all animal phyla except the Cnidaria [21,22,23]. However, attempts to use mitochondrial gene sequences to study intraspecific variation, discriminate morphospecies, or reveal phylogenetic relationships among ciliates have been restricted to relatively few taxa, e.g., *Frontonia*, *Paramecium*, *Tetrahymena*, *Carchesium polypinum*, and *Miamiensis avidus* [24,25,26,27,28,29,30,31,32,33,34]. This could be attributed to the general disadvantage of rapidly evolving markers, i.e., primer design can be elaborate and, once developed, applications of the primers are often restricted to one species only. Fortunately, a few available primer sets show great potential for the amplification of more variable DNA fragments, e.g., CO1 and ITS1-5.8S-ITS2-5′LSU rDNA, in certain species belonging to classes Oligohymenophorea and Spirotrichea [24,30,35,36].

Ciliates show a wide distribution with members present in various biotopes such as freshwater, brackish water, marine, and terrestrial environments, from the tropics to polar regions [37,38,39,40,41,42,43,44]. However, due to the imbalance of regional research and insufficient sampling, little is known about the geographical distribution pattern and genetic diversity. Nowadays, it is well known that sequencing technology can provide rich data, which is conducive to the study of ciliates biogeography and population genetics [45]. *Euplotes* may be one of the best studied ciliate genera from a morphological perspective. Furthermore, as a speciose genus, *Euplotes* includes more than 100 nominal species [38,46,47,48], which provides a good opportunity to study sequence variation at the genus and species level and to explore the availability and utility of molecular markers.

Traditionally, *Euplotes* species have been identified based on their biotope, living morphology, ciliary pattern, and silver-line system. Nuclear ribosomal gene sequences, especially SSU rDNA, have also been widely used in phylogenetic analysis [46,47,48,49,50]. By contrast, there is only one report that includes information on the genetic divergence of the CO1 gene in *Euplotes*, i.e., Park et al. [35], who obtained the CO1 sequences of 47 spirotrichs, including five identified *Euplotes* species, and analyzed the intraspecific variation in two of these. Furthermore, previous studies have demonstrated that phylogenetic analyses based on a single gene marker may give biased results and recommended that at least two loci should be used [18,46,51]. Therefore, in the present study we applied two loci (SSU rDNA and CO1 DNA) to shed some light on intraspecific relationships among certain *Euplotes* species.

In order to reduce error rates caused by misidentification, we used only well-outlined species in the present study. This resulted in the obtaining of ten new CO1 sequences and four new SSU rDNA sequences from four species. Combining other data downloaded from GenBank, we constructed a data set comprising 27 species. Comparative and phylogenetic studies were performed, the main aims of which were to: (1) investigate how much nucleotide sequence divergence occurs within *Euplotes*; (2) assess whether the CO1 gene can be used as a biodiversity marker.

## 2. Materials and Methods

### 2.1. Ciliate Culture and Identification

Ten *Euplotes* populations were collected from various habitats in China (Table 1). Morphological methods (e.g., live observation, protargol and wet silver nitrate staining) were performed to determine their species identity [52].

### 2.2. DNA Extraction

A single, live cell of each population was isolated under a stereomicroscope, washed five times in sterilized ambient water, and transferred to 1.5 ml microcentrifuge tubes. Genomic DNA was then extracted using a DNeasy Blood and Tissue Kit (Qiagen, Hilden, Germany) following the manufacturer’s instructions.

### 2.3. Amplification and Sequencing

Amplification of the SSU rRNA and CO1 genes were performed with primer sets according to [53] and [35], respectively, i.e., 18SF: 5′-AACCTGGTTGATCCTGCCAGT-3′; 18SR: 5′- TGATCCTTCTGCAGGTTCACCTAC-3′; CiCO1 Fv2: 5′-GWT GRG CKA TGA TYA CAC C-3′; CiCO1 Rv2: 5′-ACC ATR TAC ATA TGA TGW CC-3′. The PCR amplifications were performed using Q5 Hot Start High-Fidelity 2x Master Mix DNA Polymerase (NEB, Ipswich, MA) with the following protocol: one cycle of initial denaturation at 98 ℃ for 30 s, followed by 18 cycles of amplification (98 ℃, 10 s; 69 ℃–51 ℃ touch down, 30 s; 72 ℃, 1 min (CO1, 30 s)) and another 18 cycles (98 ℃, 10 s; 51 ℃, 30 s; 72 ℃, 1 min (CO1, 30 s)), with a final extension of 72 ℃ for 5 min. The PCR products were detected using agarose gel and then sequenced bidirectionally by the Tsingke Biological Technology Company (Qingdao, China). Contigs were assembled by Seqman (lasergene DNAStar).

### 2.4. Sequence and Pairwise Genetic Distances Analyses

All new nucleotide sequences have been submitted to the GenBank. Sequences of SSU and CO1 gene were aligned with the GUIDANCE2 algorithm (http://guidance.tau.ac.il/ver2/; 10 August 2021), and BioEdit 7.0 [54] was used to manually modify the resulting alignment by trimming both ends, leaving a consensus matrix of 1865 nucleotide positions (SSU rDNA) and 478 bp (CO1 gene). Pairwise genetic distance results were also obtained by BioEdit 7.0. Analysis of haplotype diversity and its sampling variance (SD), nucleotide diversity, and variable nucleotide positions was done with DnaSP v5.10.01 [55].

### 2.5. Phylogenetic Analyses

To infer the phylogenetic position of the four *Euplotes* species, 99 gene sequences retrieved from the GenBank database, including 19 mitochondrial CO1 sequences (*Caryotricha marina* and *Diophrys scutum*, as outgroups) and 80 SSU rDNA sequences (*Caryotricha marina* and *Diophrys scutum* as outgroups), and the 14 new sequences obtained in this study, were used. The final alignment used for phylogenetic analyses included 2033 sites for SSU rDNA of 52 species; 1865 sites for SSU rDNA of ten species with CO1 sequences available from the same isolates; and 478 sites for CO1 gene of ten species. A maximum likelihood (ML) analysis with 1000 bootstrap replicates was performed on the CIPRES Science Gateway, applying the GTRGAMMA model [56] using RAxML-HPC2 on XSEDE 8.2.12 [57]. A Bayesian inference (BI) analysis was carried out with MrBayes on XSEDE 3.2.7a [58] on the CIPRES Science Gateway using the model GTR+I+G, selected by AIC in MrModeltest 2.2 [59]. Markov chain Monte Carlo (MCMC) simulations were run for 1,000,000 generations with sampling every 100 generations and a burn-in of 1000 trees. MEGA 5 [60] was used to visualize the tree topologies.

## 3. Results

Sequences of the nuclear SSU rDNA and CO1 fragments were newly obtained from10 isolates of the *Euplotes* species (see Table 1 for accession numbers). All positions containing gaps and missing data were eliminated.

### 3.1. SSU rDNA Sequence Analysis

**Figure 1:**
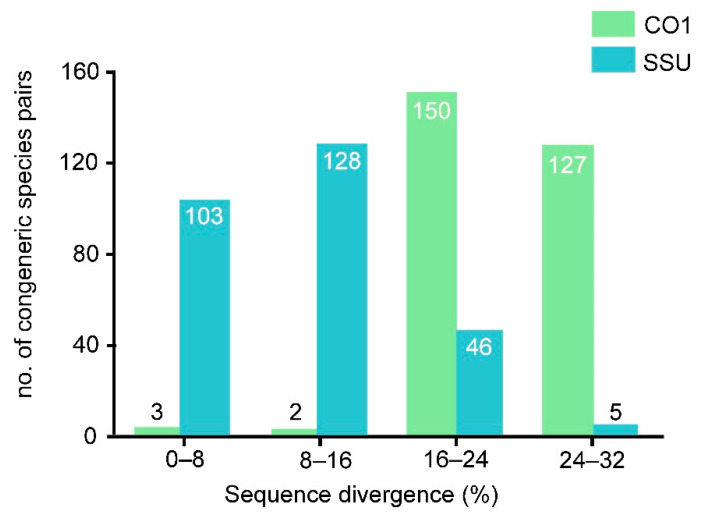
SSU rDNA and CO1 sequence divergences for 282 congeneric pairs of *Euplotes* species.

The SSU rDNA sequences of ten populations (Ev8-11, Er1-3, Eb1-2, En) were between 1480 and 1905 nucleotides long, and GC content was in the range of 43.31–46.15% (44.67 ±1.08%); the mean interspecific sequence divergence was 15.83% and ranged from 7.7–29.1% (Table S1). The SSU rDNA sequences of all 27 populations assigned to the ten species were analyzed here. Among the 282 species pairs, more than half (ca. 63.5%) showed greater than 8% sequence divergence (Figure 1; Table 2). Within *Euplotes vannus*, almost all sequences were identical except for that of isolate A764, which was separated by ca. 19% divergence. The mean sequence variation within *E. vannus* was 3.73%, which was significantly higher than those in *Euplotes raikovi* (ca. 0.93%) and *E. minuta* (0.13%). By contrast, the two populations of *Euplotes balteatus* had 8.3% gene divergence (Table S1). The intraspecific haplotype diversity value was Hd = 0.618 (SD = 0.164) in *E. vannus*, which was similar to those of *E. minuta* and *E. raikovi* (Table 3). In contrast, every studied isolate had its own haplotype in *E. balteatus*. Both *E. minuta* and *E. raikovi* showed very low levels of nucleotide diversity (less than 0.0005; Table 3). The 11 populations of *Euplotes vannus* can be divided into four groups, with low intragroup diversity (less than 1%) but high intergroup divergence (up to 18.9%).

### 3.2. CO1 Sequence Analysis

A portion of 478 bp from the mitochondrial CO1 gene of the ten species was used for the sequence analyses. The GC content was in the range of 30.13–41.21% (36.37 ± 3.1%; Table 1) based on 27 sequences including the ten new ones. Within *Euplotes*, CO1 sequence divergence among 282 species pairs ranged from a low of 0.0% to a high of 30.0% (Table S1), with a mean interspecific divergence of 23.1%, and 98.2% of species pairs showing greater than 16% sequence divergence (Figure 1, Table 2). Within *Euplotes vannus*, the mean sequence divergence was 13.7% and ranged from 0–21.4%. *Euplotes minuta* and *Euplotes raikovi* had a mean intraspecific divergence of 2.2% and 1%, respectively, and ranged from 0–5.5%. *Euplotes balteatus* showed a high level of intraspecific divergence (22.9%). The haplotype diversity value of *Euplotes minuta* was Hd = 0.4 (SD = 0.237), the lowest in the four studied species. In contrast, almost every studied strain had its own haplotype in *E. vannus* and *E. balteatus*, the latter revealing the highest level of nucleotide diversity (Table 3). *Euplotes vannus* contained five groups with low intragroup diversity (less than 3%) but high intergroup divergence (ranging from 9.0% to 21.2%).

### 3.3. Phylogenetic Analysis Based on Comparison of SSU rDNA Sequences

The phylogenetic trees constructed using two different methods (ML and BI) showed a similar topological structure, hence only ML trees are presented here.

**Figure 2:**
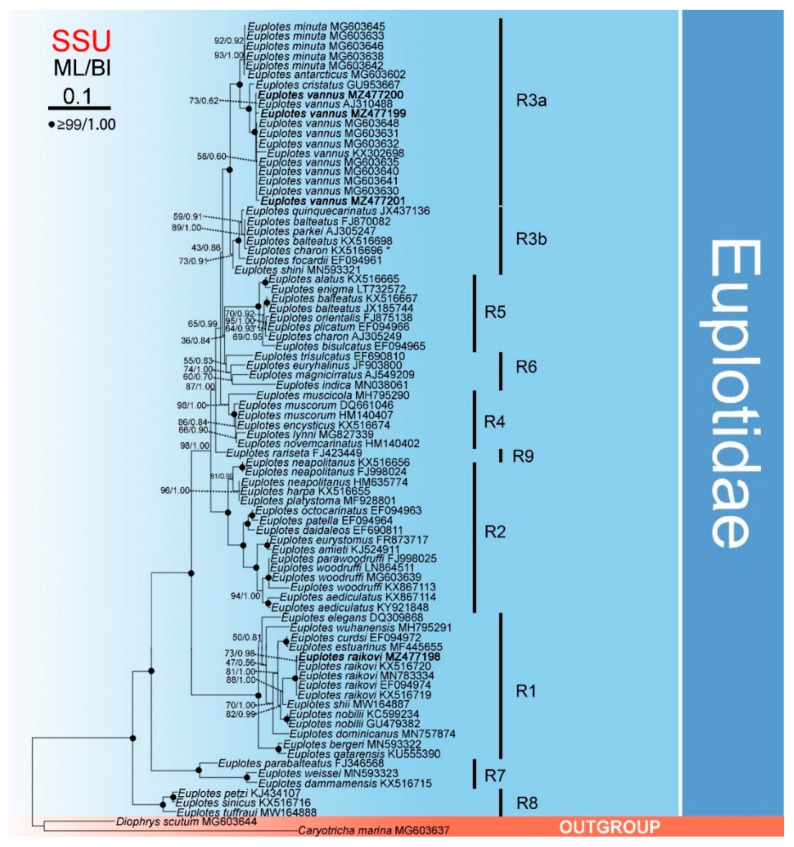
Maximum likelihood (ML) tree inferred from SSU rDNA sequences showing nodal support for ML and BI analyses. Newly sequenced species are shown in bold. A hyphen (−) reflects disagreement in topology between the BI and ML trees. R1–9 represent different clades. The scale bar corresponds to 10 substitutions per 100 nucleotide positions. * misidentification, should be *Euplotes balteatus*.

The phylogenetic trees, based on a comparison of the SSU rDNA fragments (Figure 2) and constructed for 82 *Euplotes* populations (with *Diophrys scutum* and *Caryotricha marina* as outgroup), revealed the monophyly of the genus *Euplotes* as all 52 species for which sequence data are available grouped together with maximum support. The genus was divided into nine clades (marked on the tree as R1 to R9). *Euplotes rariseta* appeared as a separate branch (R9), while the other isolates from different species formed eight clades on both trees. Among these clades, R1–5 were formed by five or more species each. Isolates belonging to *E. vannus*, *E. raikovi*, and *E. nobilii* formed monophyletic clusters for each species within the clades, while isolates of E. blateatus were distributed among two distinct clades (R3 and R5). The cluster of E. vannus grouped with E. cristatus, E. minuta, and E. antarcticus, forming a well-supported subclade (R3a). The two earliest branching clades were composed of three recently published species each.

**Figure 3:**
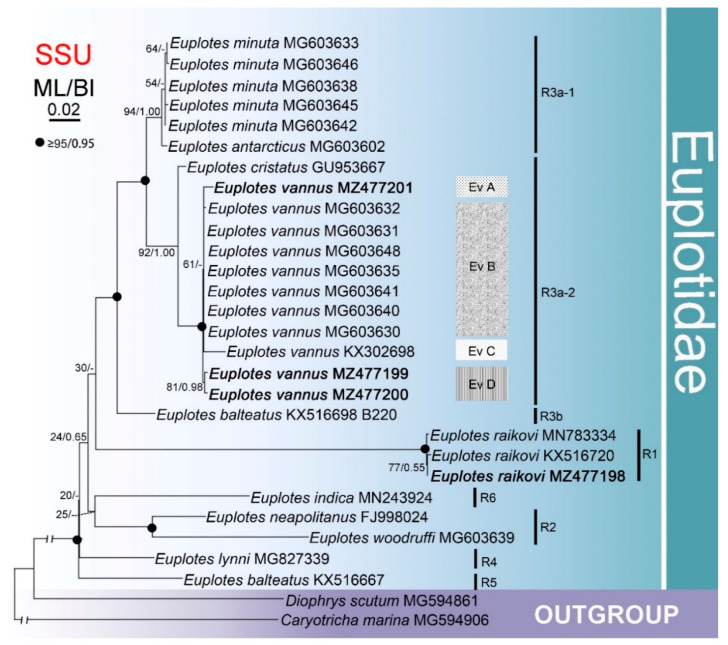
Maximum likelihood (ML) tree inferred from SSU rDNA sequences of 10 *Euplotes* species that had isolates for which CO1 sequence data are also available. Nodal support is given for both ML and BI analyses. Newly sequenced species are shown in bold. A hyphen (−) reflects disagreements in topology between the BI and ML trees. R1–6 represent different clades and Ev A–D for groups of *Euplotes vannus*. The scale bar corresponds to two substitutions per 100 nucleotide positions.

The phylogenetic trees for the SSU rDNA fragments of only ten *Euplotes* species with CO1 sequences available from the same isolates (with *Diophrys scutum* and *Caryotricha marina* as the outgroup) had a similar topology (Figure 3) to the ribosomal tree that included all 52 species for which sequence data are available (Figure 2). The isolates of these ten *Euplotes* species formed six clusters (R1–6), four of which consisted of one or two species each. R3 was composed of three subclades, viz. *Euplotes minuta* + *E. antarcticus*, *Euplotes vannus* + *E. cristatus*, and the Hong Kong population of *E. balteatus*.

Although *Euplotes vannus* formed a well-separated cluster, the SSU rDNA tree revealed the existence of four intraspecific groups, namely Ev A, Ev B, Ev C, and Ev D, corresponding to the divergences demonstrated previously. Ev A and Ev C each contained one China isolate, viz. from the Shenzhen and Qingdao regions, respectively. Ev B was composed of five isolates from Incheon, South Korea, one from Jeju-do Island, South Korea, and one from New York, USA. Ev D consisted of two Qingdao isolates.

### 3.4. Phylogenetic Analysis Based on Comparison of Partial CO1 Sequences

**Figure 4:**
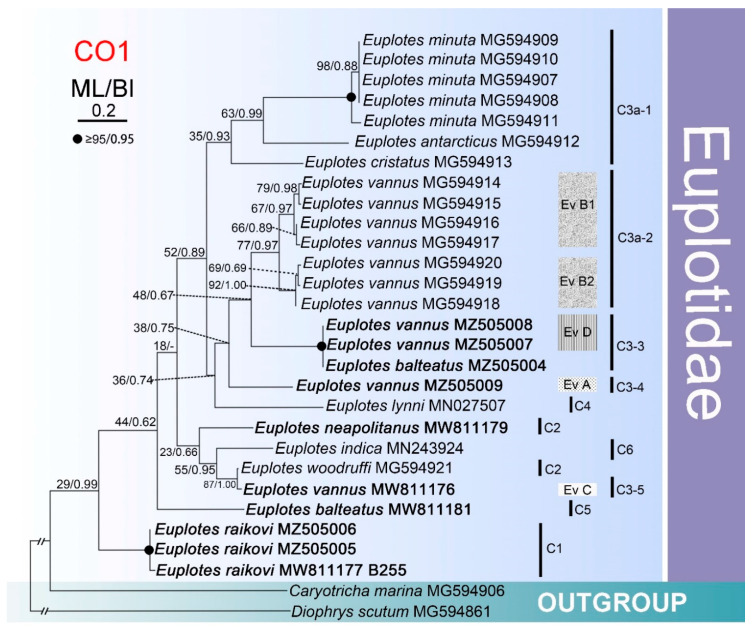
Maximum likelihood (ML) tree inferred from mitochondrial cytochrome *c* oxidase subunit 1 (CO1) nucleotide sequences of ten *Euplotes* species showing nodal support for ML and BI analyses. Newly-sequenced species are shown in bold. Disagreements between ML and BI are shown by hyphens. C1–6 represent different clades and Ev A–D for groups of *Euplotes vannus*. The scale bar corresponds to 20 substitutions per 100 nucleotide positions.

The ML and BI trees based on the CO1 gene sequences of ten *Euplotes* species (27 isolates) had similar topologies, therefore only the ML tree is shown here (Figure 4). However, the topology of this tree is somewhat different to that of the equivalent SSU rDNA tree (Figure 3). For easier orientation, clades on the phylogenetic tree were numbered as in the SSU rDNA tree and differed only in the letter (R—for SSU rDNA (Figure 2 and Figure 3), C—for CO1 (Figure 4)). In total, ten clusters were recovered (Figure 4).

Both in the ribosomal and mitochondrial trees, *E. balteatus* (L20) appeared as a separate branch (C5), which did not group with *E. balteatus* (B220). In the case of the CO1 tree, the earliest branching clade (C1) consisted of only isolates of *Euplotes raikovi*, which also formed a long-branched and well-supported cluster (R1) in the SSU rDNA tree. Another well-supported cluster corresponded to *Euplotes minuta* which showed a close relationship with *E. antarcticus*, as in the rDNA tree (Figure 2, Figure 3 and Figure 4). Unlike in the SSU rDNA tree where all isolates of *Euplotes vannus* clustered together with maximal support, in the CO1 tree they were divided into five groups (Ev A, Ev B1, Ev B2, Ev C, Ev D) of which four grouped together but with weak support. Another Qingdao isolate of *Euplotes vannus* (A764) nested into a separate clade and had a closer relationship with *E. woodruffi*, a form of which is sister to *E. neapolitanus* in the ribosomal tree (Figure 3). It is noteworthy that two Qingdao isolates of *E. vannus* (A940, A844) formed a fully supported cluster with the Hong Kong isolate of *E. balteatus*, as they had identical sequences and were sister to a cluster formed by isolates of *E. vannus* from South Korea and the USA.

Other differences between the CO1 and SSU rDNA trees include the positions of particular subclades or species, e.g., *E. raikovi* (C1), *E. neapolitanus* and *E. woodruffi*, and *E. cristatus*.

## 4. Discussion

### 4.1. Genetic Variability within Euplotes in Comparison with Certain Other Genera

In the present work, we revealed the level of genetic variability of two genomic fragments within the genus *Euplotes* by comparing our results with the background of several other ciliate genera (for example *Paramecium*, *Diophrys*, *Pseudokeronopsis*). The mean intraspecific divergences of *Euplotes* spp. differ greatly, ranging from 0.09% to 0. 83% for SSU rDNA, and 0.1% to 22.9% for CO1, which is the greatest variability of the studied mitochondrial fragments in ciliates reported to date [18,30,31,32,35]. The mean interspecific variability for CO1 within *Euplotes* is generally larger than that of *Diophrys* and *Uronychia* (Tables S1 and S2), as well as some species of *Paramecium* [24]. At present, we cannot explain exactly why *Euplotes* shows such a great divergence, but one possibility is that its species differentiated earlier and underwent a relatively longer period of evolution.

Compared with the SSU rDNA data, the mitochondrial data revealed considerably higher sequence divergences within and among the investigated *Euplotes* species (Table 2 and Table S1). Moreover, the two studied fragments revealed intraspecific variability within *Euplotes vannus*, but the mitochondrial sequences showed a clearer differentiation of the isolates (Figure 2, Figure 3 and Figure 4; Table S1).

### 4.2. The Genus Euplotes Contains Both Polyphyletic and Monophyletic Species

Based on all the *Euplotes* species for which SSU rDNA sequence data are available, the present analysis corroborates the monophyly of *Euplotes* [44,46,47,48,49,61]. Nevertheless, our findings revealed more well-supported phylogenetic clades than previous phylogenetic studies [50,62,63]. In part, this can be attributed to the increased taxon sampling, which improved analytic resolution and helped to recover phylogenetic relationships among related species in the ribosomal tree (Figure 2 and Figure 3). For example, the phylogenetic positions of *Euplotes raikovi*, *E. indica*, and *E. lynni* remain uncertain in Figure 3, but their phylogenies were resolved in Figure 2.

Comparative analyses of two genome fragments, using 27 isolates belonging to ten species, revealed significant sequence variability and a range of degrees of intraspecific differentiation in particular species (as described above). Based on the obtained trees, *E. raikovi* forms a monophyletic cluster, which is consistent with its morphological classification [37,47]. *Euplotes aediculatus* and *E. nobilii* also form monophyletic clusters on the SSU rDNA tree as in previous studies [43]. These three species have stable morphological traits among populations, even though they have wide geographic distributions [46,47,64]. This congruence between morphological and genetic discrimination could be explained by the fact that they are well-differentiated species.

A few morphologically indistinguishable species do not correspond to the species boundaries determined by genetics. Although more isolates were used for the analysis in the present study, *E. vannus* was revealed to be monophyletic, based on the analyses of the SSU rDNA sequence data comparison, as reported previously [35]. In contrast, the CO1 analysis revealed *E. vannus* to be polyphyletic. Similar to a previous study [35], five strains of *E. minuta* formed a monophyletic cluster in the mitochondrial tree, but this was not supported in the ribosomal tree.

In contrast to the above-mentioned species, *E. balteatus* is polyphyletic. The genetic diversity of *E. balteatus* isolates has been studied twice using the ribosomal molecule [50,61], however it has never been investigated using the CO1 fragment. The present study revealed a significant sequence divergence between two populations in both rDNA and CO1 loci, which infers the presence of putative cryptic species, as suggested by Barth et al. [24].

### 4.3. Utility of SSU rDNA and CO1 as Biodiversity Markers

An important task for evolutionary biologists is to identify and determine the systematic positions of related taxa using information derived from their genomes. The molecular phylogeny of ciliates is mainly based on SSU rDNA sequence data, and this has helped to resolve a great number of systematic problems [6,9]. However, while some authors have detected higher interspecific variation using SSU rDNA [8,10,11,12,13,14], others have obtained no or low sequence variability between certain congeners, or even between different genera, for example, in *Pseudourostyla* [15], *Pseudouroleptus* [16], *Tetrahymena* [17], *Pseudokeronopsis* [18], *Euplotes* [35], and *Paramecium* [65]. This indicates the low resolution of SSU rDNA as a species discriminator. Therefore, another gene marker is urgently required to identify species. The mitochondrial cytochrome *c* oxidase 1 (CO1) gene shows a high evolutionary rate and high copy number per cell [66]. Moreover, this DNA fragment has been shown to be effective in identifying many metazoans [20,21,22,23]. These render the CO1 gene as a marker of choice. In the last 15 years, several attempts have been made to use mitochondrial sequences to study genetic variation in ciliates. According to these studies, mitochondrial data revealed substantially higher divergences within and between the investigated species and could also serve as a tool to study population genetics and identify species, possibly even new taxa. However, our study obtained different results for different species. The nucleotide divergence within *Euplotes vannus* and *E. balteatus* is extremely high (up to 23%), whereas in *E. raikovi* it is rather low (0–1.5%). The same pattern was also found using the SSU rDNA sequence. Based on the threshold values for species identities suggested by barcoding studies, high divergences indicate the presence of several cryptic species, though there is no single fixed threshold rate that can be applied to all ciliates [36]. Extremely low levels of divergence within species in diverse groups of organisms do not allow for the separation of populations in the same species, but enable the identification of known species [17,35]. Consequently, the application of the CO1 gene in ciliates should be cautious owing to the intrinsic limitations of the mtDNA due to its fast evolutionary rate, possible horizontal gene transfer, and the paucity of sequences in the CO1 barcode database.

### 4.4. Biogeography of Certain Species within Euplotes

Common views on the global geographic distribution of microeukaryotes can be divided into two schools. Microorganisms (including protists, bacteria, fungi, etc.) are generally considered to be globally distributed, as stated by Beijerinck (1913) [67], i.e., “everything is everywhere, but the environment selects”. This can be interpreted as: microorganisms will appear wherever their biological needs are met [45,68]. Foissner proposed a moderate endemicity model, suggesting that specific ciliate species exist in a limited geographical range [69]. Evidence suggests that, with a large number of species reported worldwide and the improvement of molecular marker resolution, the distribution pattern of eukaryotic microorganisms appears to be both global and localized [30,70].

Morphological studies have revealed that some *Euplotes* species, such as *E. vannus* and *E. minuta*, are abundant and widely distributed (e.g., found in both the eastern and western coasts of the Pacific Ocean). *Euplotes raikovi* is also found in both the western coast of the Pacific Ocean and the Mediterranean Sea. In contrast, other species seem to be distributed only locally, e.g., *E. antarcticus* and *E. forcardi* are found only in Antarctica [35,44]. Thus, there is support for both hypotheses based on the distribution of ciliates.

The current available molecular data do not demonstrate a clear connection between geographical and genetic distances. With regard to *E. vannus*, there were no significant genetic differences among populations from distant localities based on SSU rDNA sequence data. In contrast, on the CO1 tree, South Korean and American populations clustered together, and the two populations (Qingdao and Shenzhen) from China branched separately. Likewise, neither *E. minuta* nor *E. raikovi* showed a geographical pattern. Isolates of *E. minuta* from South Korea were assigned to different groups, while four populations from the USA and South Korea had almost identical CO1 sequences (Table 1 and Table S1; Figure 4). Moreover, for *E. raikovi*, one Chinese population was more closely related to the Italian isolate than the other Chinese one. So far, whether or not *Euplotes* species show a biogeographical pattern has not yet been confirmed using genetic data.

## 5. Conclusions

Based on the analyses of newly obtained sequences, more putative cryptic species were identified in morphologically indistinguishable species. The CO1 gene presents great potential for discriminating populations within certain *Euplotes* species. Additionally, increased taxon sampling may give an opportunity to screen relationships and distribution patterns among members of this genus.

## Figures and Tables

**Table 1:** Specifications of species with both SSU rDNA and partial CO1 gene sequence data from the same isolates analyzed in this study (new sequences in bold).

Species	Geographic Origin	Isolate ID	Abbreviation	GenBank Accession Numbers		GC% of CO1	GC% of SSU
				CO1	SSU		
*Euplotes antarcticus*	King George Island, Antarctic	INHC065	Ea	MG594912	MG603602	39.54%	43.59%
*Euplotes cristatus*	Incheon, South Korea	KS008	Ecr	MG594913	GU953667	41.00%	43.75%
*Euplotes indica*	Raj Ghatpond, India	OBS2	Ei	MN243924	MN038061	32.53%	43.50%
*Euplotes lynni*	the Sanjay Lake, India	SLS1	El	MN027507	MG827339	37.16%	43.90%
*Euplotes vannus*	Incheon, South Korea	KS106	Ev1	MG594914	MG603630	36.82%	43.85%
*Euplotes vannus*	Incheon, South Korea	KS107	Ev2	MG594915	MG603631	36.61%	43.87%
*Euplotes vannus*	Jeju-do Island, South Korea	KS012	Ev3	MG594916	MG603635	35.77%	43.83%
*Euplotes vannus*	East River, New York, USA	WS052	Ev4	MG594917	MG603648	36.36%	43.85%
*Euplotes vannus*	Incheon, South Korea	KS006	Ev5	MG594918	MG603640	37.45%	43.85%
*Euplotes vannus*	Incheon, South Korea	KS074	Ev6	MG594919	MG603641	38.08%	43.85%
*Euplotes vannus*	Incheon, South Korea	KS109	Ev7	MG594920	MG603632	37.66%	43.85%
*Euplotes vannus*	Qingdao, China	A764	Ev8	**MW811176**	KX302698	34.10%	43.38%
*Euplotes vannus*	Qingdao, China	A940	Ev9	**MZ505008**	**MZ477200**	34.73%	43.91%
*Euplotes vannus*	Qingdao, China	A844	Ev10	**MZ505007**	**MZ477199**	34.73%	44.20%
*Euplotes vannus*	Shenzhen, China	C102	Ev11	**MZ505009**	**MZ477201**	34.38%	43.78%
*Euplotes woodruffi*	Songjiho Lagoon, Gangwon-do, South Korea	KS056	Ew	MG594921	MG603639	33.68%	44.81%
*Euplotes minuta*	Jeju-do Island, South Korea	KS011	Em1	MG594907	MG603633	41.21%	43.61%
*Euplotes minuta*	Busan, South Korea	KS044	Em2	MG594908	MG603638	41.21%	43.61%
*Euplotes minuta*	Laguna Beach, California, USA	WS038	Em3	MG594909	MG603645	41.21%	43.61%
*Euplotes minuta*	Laguna Beach, California, USA	WS039	Em4	MG594910	MG603646	41.21%	43.61%
*Euplotes minuta*	Chungcheongnam-do, South Korea	KS082	Em5	MG594911	MG603642	39.33%	43.61%
*Euplotes raikovi*	Zhanjiang, China	B255	Er1	**MW811177**	KX516720	32.85%	46.15%
*Euplotes raikovi*	Qingdao, China	A714	Er2	**MZ505005**	MN783334	32.64%	45.51%
*Euplotes raikovi*	Adriatic Coast, Italy	A841	Er3	**MZ505006**	MZ477198	32.64%	45.81%
*Euplotes balteatus*	Hong Kong, China	B220	Eb1	**MZ505004**	KX516698	34.73%	44.85%
*Euplotes balteatus*	Daya Bay, Huizhou, China	L20	Eb2	**MW811181**	**KX516667**	34.31%	45.79%
*Euplotes neapolitanus*	Shenzhen, China	L10	En	**MW811179**	FJ998024	30.13%	43.31%

**Table 2:** Mean and standard deviation (s.d.) of the percentage sequence divergences of CO1 or SSU rRNA genes for congeneric species pairs within three genera. (The percentage of sequence divergence estimates falling in a particular range is also shown. *n* indicates the number of congeneric pairs examined in each genus).

Genus	Gene	n	Mean	s.d.	Sequence Divergence (%)			
					0–8	8–16	16–24	24–32
*Euplotes*	CO1	282	23.1	3.4	1.1%	0.7%	53.2%	45.0%
	SSU	282	10.6	5.7	36.5%	45.4%	16.3%	1.8%
*Diophrys*	CO1	21	18.8	0.4	0	0	100.0%	0
*Uronychia*	CO1	1	n/a	n/a	0	0	100.0%	0

**Table 3:** Variability of the SSU rDNA and CO1 fragments in four species of *Euplotes* with more than two isolates each.

Species	SSU rDNA				CO1 mtDNA			
	Number of Sequences	Number of Haplotypes	Haplotype Diversity (SD)	Nucleotide Diversity	Number of Sequences	Number of Haplotypes	Haplotype Diversity (SD)	Nucleotide Diversity
*Euplotes vannus*	11	5	0.618 (0.164)	0.00177	11	10	0.982 (0.046)	0.13551
*Euplotes minuta*	5	3	0.700 (0.218)	0.00044	5	2	0.400 (0.237)	0.02176
*Euplotes raikovi*	3	2	0.667 (0.314)	0.00038	3	2	0.667 (0.314)	0.00976
*Euplotes balteatus*	2	2	1.000 (0.500)	0.05039	2	2	1.000 (0.500)	0.22803

## Data Availability

Sequences data are available in GenBank. The datasets used and/or analyzed during the current study are available from the corresponding author on reasonable request.

## Supplementary Materials

The following are available online at https://www.mdpi.com/article/10.3390/microorganisms9112204/s1, Table S1: Pairwise genetic distances between the investigated *Euplotes* species, Table S2: Pairwise genetic distances and different base pairs between the investigated *Diophrys* and *Uronychia* species for the CO1 gene.
