# Supplementary material for: Genetic Diversity and Phylogeny of the Genus Euplotes (Protozoa, Ciliophora) Revealed by the Mitochondrial CO1 and Nuclear Ribosomal Genes
Source: Microorganisms. 2021 Oct 22;9(11):2204. doi: 10.3390/microorganisms9112204 (PMC8624429; doi:10.3390/microorganisms9112204)
Supplement: Supplementary file 1 [file microorganisms-09-02204-s001.zip › microorganisms-1393652-supplementary.pdf]

microorganisms

MDPI

Supplementary Material

Table S1. Pairwise genetic distances between the investigated *Euplotes* species. Abbreviations of the clonal lineages are as in Table 1. Lower left, partial mitochondrial CO1 distances; upper right, nuclear SSU rDNA distances.

Seq->	Ea	Ec	Ei	El	Ev1	Ev2	Ev3	Ev4	Ev5	Ev6	Ev7	Ev8	Ev9	Ev10	Ev11	Ew	Em1	Em2	Em3	Em4	Em5	Er1	Er2	Er3	Eb1	Eb2	En
Ea	-	3.3	10.1	8.0	4.5	4.5	4.5	4.5	4.5	4.5	4.6	20.4	4.7	4.9	5.1	10.6	0.2	0.2	0.3	0.3	0.3	16.9	15.8	15.8	5.3	9.8	7.7
Ec	22.9	-	11.7	9.7	2.5	2.5	2.5	2.5	2.5	2.4	2.6	19.4	2.5	2.7	2.9	11.6	3.3	3.3	3.3	3.3	3.4	16.2	15.1	15.1	6.8	10.0	8.6
Ei	30.0	27.9	-	8.7	12.2	12.2	12.2	12.2	12.2	12.3	12.2	25.0	12.3	12.6	12.6	12.7	10.2	10.2	10.3	10.3	10.3	19.7	18.6	18.6	11.6	13.6	10.3
El	25.6	22.0	26.0	-	10.2	10.2	10.2	10.2	10.2	10.2	10.2	23.2	10.3	10.6	10.4	12.1	8.1	8.1	8.1	8.1	8.2	18.1	16.9	16.9	9.4	12.5	8.9
Ev1	24.1	19.9	25.6	22.6	-	0	0	0	0	0	0.1	18.6	0.5	0.7	0.9	11.8	4.4	4.4	4.5	4.5	4.5	16.9	15.8	15.8	7.6	11.2	9.3
Ev2	23.9	20.1	25.4	22.4	1.1	-	0	0	0	0	0.1	18.6	0.5	0.7	0.9	11.8	4.4	4.4	4.5	4.5	4.5	16.9	15.8	15.8	7.6	11.2	9.3
Ev3	23.9	19.7	26.2	23.1	3.2	3.0	-	0	0	0	0.1	18.6	0.5	0.7	0.9	11.8	4.4	4.4	4.5	4.5	4.5	16.9	15.8	15.8	7.6	11.2	9.3
Ev4	24.1	19.7	26.2	23.5	4.0	3.8	1.3	-	0	0	0.1	18.6	0.5	0.7	0.9	11.8	4.4	4.4	4.5	4.5	4.5	16.9	15.8	15.8	7.6	11.2	9.3
Ev5	22.9	20.8	25.6	22.6	10.1	9.9	9.0	10.3	-	0	0.1	18.6	0.5	0.7	0.9	11.8	4.4	4.4	4.5	4.5	4.5	16.9	15.8	15.8	7.6	11.2	9.3
Ev6	22.9	21.0	25.6	22.4	10.3	10.1	9.7	10.9	1.5	-	0.1	18.6	0.4	0.6	0.8	11.8	4.5	4.5	4.5	4.5	4.6	16.9	15.8	15.8	7.7	11.2	9.4
Ev7	23.7	20.8	25.8	22.9	10.3	10.1	9.7	10.9	1.9	1.7	-	18.7	0.6	0.8	1.0	11.9	4.5	4.5	4.6	4.6	4.7	16.9	15.8	15.8	7.7	11.1	9.3
Ev8	24.5	22.9	18.0	22.2	20.8	20.6	21.0	20.8	20.6	21.2	21.0	-	18.8	19.0	18.9	24.8	20.4	20.4	20.5	20.5	20.5	29.1	28.0	28.0	22.4	23.8	23.7
Ev9	23.5	20.6	24.7	24.1	16.8	16.8	16.2	16.4	18.3	18.3	17.8	21.4	-	0.6	1.0	12.0	4.7	4.7	4.7	4.7	4.8	17.1	16.0	16.0	7.9	11.4	9.5
Ev10	23.5	20.6	24.7	24.1	16.8	16.8	16.2	16.4	18.3	18.3	17.8	21.4	0	-	1.3	12.1	4.8	4.8	4.9	4.9	4.9	17.0	16.1	16.2	7.7	11.2	9.7
Ev11	24.3	22.0	24.6	21.2	18.5	17.8	18.0	17.6	17.8	18.7	18.0	19.3	17.6	17.6	-	12.3	5.1	5.1	5.1	5.1	5.2	17.3	16.1	16.2	8.4	11.6	9.9
Ew	24.1	22.9	17.4	21.8	19.9	19.7	20.6	20.3	20.6	21.2	21.0	1.7	21.4	21.4	19.1	-	10.7	10.7	10.7	10.7	10.8	18.4	17.3	17.3	11.8	14.3	8.3
Em1	23.3	23.9	29.3	23.5	24.5	24.3	24.9	24.7	24.7	24.5	24.3	23.7	22.6	22.6	23.1	24.1	-	0	0.1	0.1	0.2	17.0	15.8	15.9	5.4	9.8	7.8
Em2	23.3	23.9	29.3	23.5	24.5	24.3	24.9	24.7	24.7	24.5	24.3	23.7	22.6	22.6	23.1	24.1	0	-	0.1	0.1	0.2	17.0	15.8	15.9	5.4	9.8	7.8
Em3	23.3	23.9	29.3	23.5	24.5	24.3	24.9	24.7	24.7	24.5	24.3	23.7	22.6	22.6	23.1	24.1	0	0	-	0.2	0.2	17.0	15.9	15.9	5.5	9.9	7.8
Em4	23.3	23.9	29.3	23.5	24.5	24.3	24.9	24.7	24.7	24.5	24.3	23.7	22.6	22.6	23.1	24.1	0	0	0	-	0.1	17.0	15.9	15.9	5.5	9.9	7.8
Em5	23.3	24.5	30.0	23.5	24.9	24.7	24.9	24.7	26.0	25.8	25.8	23.3	22.6	22.6	23.1	23.5	5.5	5.5	5.5	5.5	-	17.1	15.9	16.0	5.5	9.9	7.9
Er1	23.7	25.4	24.9	24.3	24.7	25.2	24.9	24.7	24.9	25.2	24.7	19.9	24.1	24.1	22.2	20.8	27.7	27.7	27.7	27.7	25.4	-	1.4	1.3	15.4	15.2	16.6
Er2	23.7	25.4	24.5	23.7	24.7	25.2	24.9	24.5	24.5	24.7	24.3	19.5	24.1	24.1	22.0	20.3	27.5	27.5	27.5	27.5	24.5	1.5	-	0.1	16.3	16.1	15.5
Er3	23.7	25.4	24.5	23.7	24.7	25.2	24.9	24.7	24.5	24.7	24.3	19.5	24.1	24.1	22.0	20.3	27.5	27.5	27.5	27.5	24.5	1.5	0	-	16.3	16.2	15.5
Eb1	23.5	20.6	24.7	24.1	16.8	16.8	16.2	16.4	18.3	18.3	17.8	21.4	0	0	17.6	21.4	22.6	22.6	22.6	22.6	22.6	24.1	24.1	24.1	-	8.3	8.8
Eb2	23.3	23.5	27.2	23.9	23.5	23.1	22.2	22.2	21.6	22.0	22.2	21.8	22.9	22.9	21.6	21.4	24.5	24.5	24.5	24.5	23.5	22.2	21.8	21.8	22.9	-	11.8
En	23.5	23.5	20.8	23.3	18.5	18.5	28.7	20.3	19.9	20.6	20.3	15.9	19.9	19.9	20.3	14.9	23.9	23.9	23.9	23.9	24.1	21.2	20.8	20.8	19.9	18.7	-

Table S2. Pairwise genetic distances and different base pairs between the investigated *Diophrys* and *Uronychia* species for CO1 gene.

**Seq->**	**D_o64**	**Do65**	**D_o66**	**D_s57**	**D_s58**	**D_s59**	**D_s60**	**D_s61**	**D_s62**	**D_s63**	**U_b69**	**U_s68**
D_o64	-	0	1	91	91	91	90	89	86	91	117	112
D_o65	0.0	-	1	91	91	91	90	89	86	91	117	112
D_o66	0.3	0.3	-	91	91	91	90	89	86	90	117	111
D_s57	19.1	19.1	19.1	-	0	0	1	2	21	61	92	115
D_s58	19.1	19.1	19.1	0.0	-	0	1	2	21	61	92	115
D_s59	19.1	19.1	19.1	0.0	0.0	-	1	2	21	61	92	115
D_s60	18.9	18.9	18.9	0.3	0.3	0.3	-	1	20	60	91	114
D_s61	18.7	18.7	18.7	0.5	0.5	0.5	0.3	-	20	59	91	114
D_s62	18.0	18.0	18.0	4.4	4.4	4.4	4.2	4.2	-	63	97	114
D_s63	19.1	19.1	18.9	12.8	12.8	12.8	12.6	12.4	13.2	-	105	117
U_b69	24.5	24.5	24.5	19.3	19.3	19.3	19.1	19.1	20.3	20.0	-	82
U_s68	23.5	23.5	23.5	24.1	24.1	24.1	23.9	23.9	23.9	24.5	17.2	-

Abbreviations: D_s57-63 for seven isolates of *Diophrys scutum*; D_o64-66 for three isolates of *Diophrys oligothrix*; U_b69 for *Uronychia binucleata*; U_s68 for *Uronychia setigera*.
